# T cell effects and mechanisms in immunotherapy of head and neck tumors

**DOI:** 10.1186/s12964-023-01070-y

**Published:** 2023-03-05

**Authors:** Yizhen Xiang, Mengdan Gong, Yongqin Deng, Hongli Wang, Dong Ye

**Affiliations:** 1 Department of Otorhinolaryngology-Head and Neck Surgery, The Affiliated Lihuili Hospital, Ningbo University, Ningbo, 315040 Zhejiang China; 2 Department of Otorhinolaryngology-Head and Neck Surgery, The Affiliated People Hospital, Ningbo University, Ningbo, 315040 Zhejiang China

**Keywords:** Head and neck cancer, Major histocompatibility complex (MHC), Immunotherapy, Immune checkpoint inhibitor (ICI)

## Abstract

Head and neck tumors (HNCs) are a common tumor in otorhinolaryngology head and neck surgery, accounting for 5% of all malignant tumors in the body and are the sixth most common malignant tumor worldwide. In the body, immune cells can recognize, kill, and remove HNCs. T cell-mediated antitumor immune activity is the most important antitumor response in the body. T cells have different effects on tumor cells, among which cytotoxic T cells and helper T cells play a major killing and regulating role. T cells recognize tumor cells, activate themselves, differentiate into effector cells, and activate other mechanisms to induce antitumor effects. In this review, the immune effects and antitumor mechanisms mediated by T cells are systematically described from the perspective of immunology, and the application of new immunotherapy methods related to T cells are discussed, with the objective of providing a theoretical basis for exploring and forming new antitumor treatment strategies.

Video Abstract

**Supplementary Information:**

The online version contains supplementary material available at 10.1186/s12964-023-01070-y.

## Background

Head and neck cancers (HNCs) account for 5% of malignant tumors, and are the sixth most common malignant tumors worldwide, and represent the eighth leading cause of tumor-related deaths [1–4]. According to the Global Cancer Information Network 2020, there are approximately 800,000 new cases of head and neck cancer worldwide annually, including 450,000 deaths [5]. HNCs is a multifactorial malignancy, traditional tobacco and alcohol have been considered the main risk factors for HNCs [6]. As smoking and HNCs related to smoking have declined in the last 20 years, the proportion of HNCs related to human papillomavirus (HPV) has increased annually [7]. Of the 600,000 cases of HNC worldwide, approximately 8.5 million cases are caused by high-risk HPV infection, making HPV the second most common cause of HNC after smoking [8]. Because HNCs are located in a key position of the upper respiratory tract, surgical treatment of HNCs often significantly compromises patient quality of life by affecting breathing, swallowing, speech, and even appearance [9].Despite recent advances in surgical radiotherapy (RT) and chemotherapy for HNCs, there is still no significant improvement in survival for HNC patients, especially for recurrent/metastatic HNCs (R/M HNCs), with few effective treatment options [10]. Therefore, it is urgent to explore immunotherapy for HNCs. Several studies have shown that the degree of T-cell infiltration in HNCs is closely related to improved prognosis [11–15]. An increase or decrease in the number of T cells in different compartments of the body can promote or inhibit the invasiveness and prognosis of HNCs [16–20]. T cell-related immunotherapy has become the main focus of HNC immunology research [21]. T cell-associated immunotherapy is long-lasting and less toxic than conventional surgery [22] or chemoradiotherapy. Immunological mechanisms based on T cells have produced different forms of immune therapy; the main purpose of these methods is to enhance the immune system response (especially T cells) and to suppress immune checkpoints [23] including tumor vaccines directed against tumor antigens, the use of targeted drugs directly stimulate the immune system, following direct inoculation tumor related to T cells and immune checkpoint therapy.

**Fig. 1:**
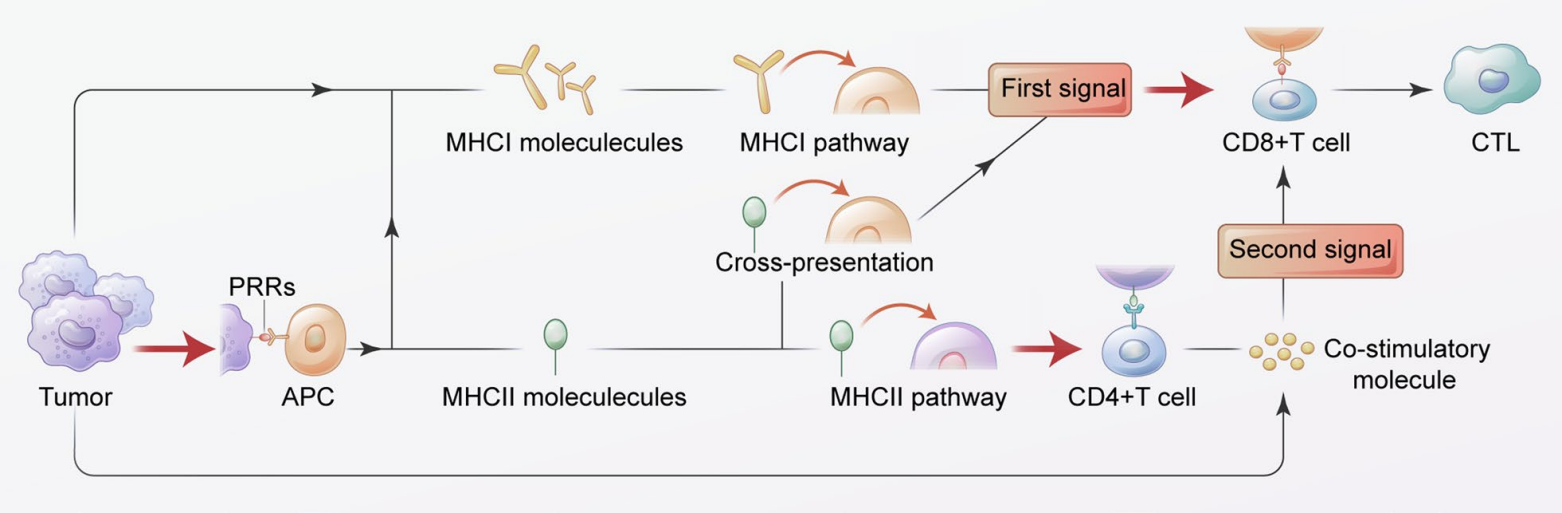
HNCs can inhibit t-cell-mediated activation of immune responses through three pathways. **1** Tumor cells can express MHC I molecules on the cell surface and form an antigenic peptide-MHC CLASS I molecular complex through the MHC I pathway, which provides the first signal for T cell activation. CD8+ T cells can initially transform into CTL after receiving the first signal **2** At the same time, tumor cells can recognize MHC class II molecules on the surface of APC cells by pattern recognition receptors (PRR). Some MHC II molecules can form the first signal through cross-presentation and participate in the initial activation of CTL cells through the endogenous antigen presentation pathway. Other MHC CLASS II molecules can form an antigenic peptide-MHC Class II molecular complex through the MHC II pathway to activate CD4+ T cells to produce costimulatory molecules, and at the same time provide a second signal for the activation of CD8+ T cells to fully activate and maintain CTL proliferation and cloning. **3** Tumor cells and APC cells can also secrete costimulatory molecules and participate in the formation of the secondary signal

In this paper, the role of T cells in the treatment of HNCs and the mechanism of immune-mediated effects mediated by T cells were systematically described from the perspective of immunology (Fig. 1), and the application of new immunotherapy methods related to T cells were analyzed, hoping to provide a theoretical basis for exploring and forming new antitumor treatment programs.

## The role of T cells in HNCs

T cells play a key role in the specific immunity of HNCs [24]. T cells can directly kill tumor cells and inhibit their proliferation, infiltration, and even tumor metastasis. T cells involved in this activity include CTL and Th cells [25]. However, tumor cells can also actively induce the production of regulatory T cells (Tregs) and other regulatory cells in the host body to resist the antitumor immune response, thus obtaining an environment suitable for proliferation and growth [26].

### The killing effect of cytotoxic T cells (CTL) and helper T cells (Th) on HNC cells

#### Direct killing effect of CTL on HNCs

All nucleated cells express major histocompatibility complex (MHC) class I molecules, and HNCs are no exception [27]. Once T cells detect the antigen peptide-MHC I complex presented by HNCs, CD8+ T cells are activated to form CTLs, thus destroying tumor cells that present target antigens. In mouse experiments, Malik et al. [28] found that CD8+ T cells isolated from vitiligo patients (patients with vitiligo overexpress CD8+ T cells) could be recognized in vitro and trigger apoptosis of mouse melanoma tumor cells [27, 29]. This experiment showed that CTL had a direct killing effect on tumor cells. CTL specifically kills tumor cells through two pathways. The first pathway is the perforin/granzyme pathway [30]. The perforin monomer can be inserted into the target cell membrane, and in the presence of calcium ions [31] multiple perforin can be polymerized into pores with larger inner diameter, so that cytotoxic proteins such as granzyme can quickly enter cells [32]. Backes et al. found in perforin-deficient mouse models that the perforin-granzyme pathway plays a dominant role in mouse and human CTL cells [33]. The second pathway is the Fas-FasL and THF-TNFR pathway or death receptor pathway. CTLs can induce target cell apoptosis by expressing FasL or secreting TNF-α and activating the intracellular signal transduction pathway involving caspases [34].

#### Killing effect of Th cells on HNCs

Th cells can kill tumor cells in two ways. First, Th can kill tumor cells directly through the cytolysis mechanism [35]. Couture et al. concluded that artificially transferred CD4+ Th in lymphocytopenia mice could not only produce granzyme B with MHCII-dependent cytotoxic activity, but also directly reject melanoma tumors through interferon (IFN)-dependent mechanism [36]. Meanwhile, Th17-polarized T cells can also directly induce tumor cell vascular necrosis by generating the angiogenic factor TNF. Conversely, Th can indirectly enhance the antitumor effect of the body by regulating CTL. Bannard et al. demonstrated that CD4+ T cells, upon recognition of MHCII, modulate antigen presenting cells (APCs) through CD40-CD40L, providing appropriate stimulation for CD8+ T cells to produce a durable immune response to cross-presented antigens on the same APC [37]. Brightman et al. concluded that without the regulatory effect of Th cells on CD8+ T, CTL cells would not be able to effectively produce memory immunity and secondary proliferation, resulting in a durable antitumor effect [38]. Doorduijn et al. [39] and Sato et al. [40]. demonstrated in different mouse experiments that Th cells can recruit and activate monocyte macrophages and CD8+ T lymphocytes through the release of cytokines and chemokines by extracelluar vesicles(EV) and induce an antitumor reaction. IFNγ-induced chemokines can recruit CTL to form an MHC II-dependent tumor-killing effect.

### Promoting the effects of regulatory T cells (Treg) on the growth of HNC

Sakaguchi et al. through a several experiments have revealed that in newborn mice thymectomy can inhibit autoimmune T cell activation and limits the associated inflammatory response by providing T cell from thymic mice, these mice achieve an autoimmune response that is dependent on T cells[41]. This experiment demonstrated the existence of a special type of T cells in the thymus that play a negative role in immune regulation [42]. In 2003, the transcription factor FoxP31 specifically expressed in Treg cells was identified in rodents and humans [43], and subsequent experiments demonstrated that FoxP31-mutated Scurfy mice spontaneously develop fatal systemic autoimmune diseases [44]. Therefore, numerous experiments have shown that Treg cells play a key role in maintaining self-tolerance and preventing various autoimmune diseases [45]. Cillo et al. found that CD137^high^ Treg enrichment was associated with poorer overall survival after analysis of 131,224 single-cell transcriptional profiles of HNCs patients and healthy donors[46].In HNCs, Tregs can bind IL-2 released by activated neighboring T cells through highly expressed receptors with high affinity for affinity for IL-2 and secrete inhibitory cytokines IL-10 and TGF-β [47], as well as directly kill effector T cells, thus maintaining immune homeostasis[48]. Early HNC cells often promote Treg production in the host body to inhibit the collective antitumor effect.

### Enhanced sensitivity of T cells to HNC chemoradiotherapy

To study the role of T cells in chemotherapy (CRT), Aurelie et al. used HPV-associated squamous cell carcinoma of the head and neck (HNSCC) as a preclinical model. In this study, the CTX and iNOS inhibitor L-NIL was used as a target to analyze differences in the immune response between the two models of radiation therapy and chemotherapy alone and combined therapy with CRT + CTX/L-NIL [49]. Combination therapy improves intratumoral T cell infiltration and tumor antigen specificity of T cells to achieve a better therapeutic effect than chemotherapy alone. The CD8+ T cell/regulatory T cell ratio increased 31.8 times in patients treated with combination immunotherapy compared to CRT alone, and tumor antigen-specific CD8+ T cells increased significantly. These data suggest that reducing intratumor Treg levels or increasing CD8+ T cell infiltration can enhance the antitumor effect of the body [50] and can increase the sensitivity of refractory tumors to CRT, thus improving the immune benefit of CRT [51–53].

Several publications have reported that the high correlation between CD8+ tumor-infiltrating lymphocytes (TIL) and CRT may be one of the key factors that mediate the effective response of CRT. Gerber et al. demonstrated that the reduction in the number of CD8+ T cells in the body would reduce the antitumor effect mediated by IFN in RT. Furthermore, chemotherapy could change the tumor microenvironment by releasing tumor antigens and promoting dendritic cell (DC) accumulation, thus improving the antitumor effects of CD8+ T cells[54]. Brakenhoff et al. analyzed the biological signals of 197 HPV-negative (nrgative advanced stage HNSCC) patients with advanced HNSCC before and after radiotherapy and chemotherapy, and the analysis results showed that high CD8+ T profile was closely associated with good prognosis[55].Recent studies have shown that TIL density and PD-L1 expression are correlated with the prognosis of HNC patients. These studies first analyzed the correlation between PD-L1 expression and CD8+ TIL density, and further investigated the relationship between these immune parameters and chemoradiotherapy sensitivity in the primary tumor region and with the prognosis of HNC patients. Studies have shown that patients with HNC with a higher DENSITY of CD8+ TIL have significantly higher disease-free survival or OS [15, 56]. Ono et al. statistically analyzed and recorded TIL density in 106 patients with HNC who received chemoradiotherapy before treatment and found that there was a positive correlation between elevated CD8+ TIL concentration and favorable response to adjuvant chemotherapy in patients with advanced hypopharyngeal cancer [57]. Meanwhile, Maimela et al. found a correlation between increased CD8+ TIL density and reduced distant metastasis of tumor cells and an increased survival rate in a large number of studies [58]. Toshihiko Kawaguchi et al. proposed that in the future, the prognostic effect of CRT in high-risk patients could be determined by assessing the density of CD8+ TIL after pretreatment, and whether adjuvant therapy (additional RT, chemotherapy or immunotherapy) would be required[59].

## Immunological mechanism of T cells in HNC therapy

**Fig. 2:**
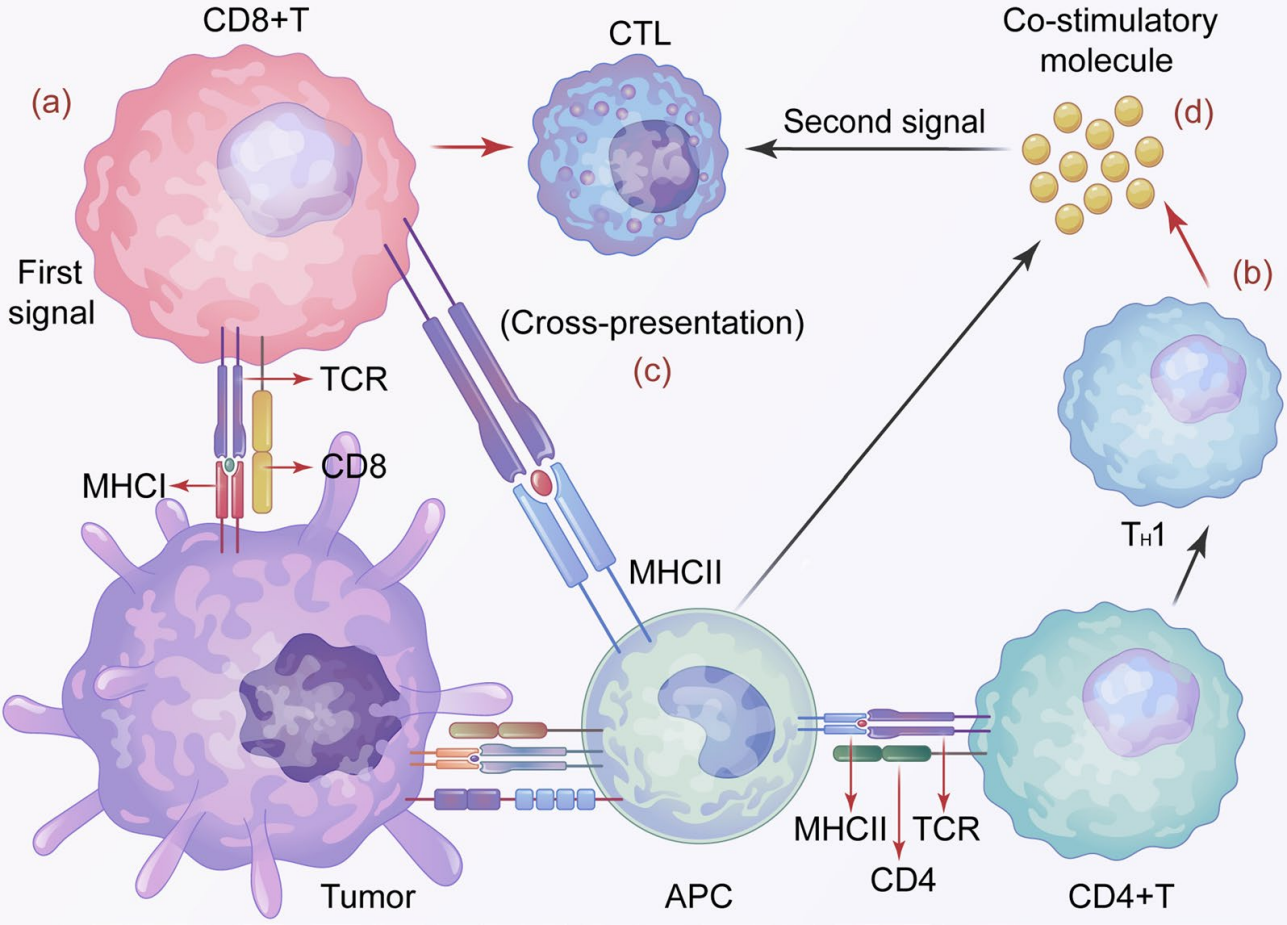
**a** Tumor cells express MHC-I molecules to provide the first signal for T cell activation through the MHC-I pathway, leading to initial activation of CD8+ T cells in CTL. **b** The APC recognizes tumor cells using pattern recognition receptors (PRRs), and then expresses MHC-II molecules and delivers them to CD4+ T cells, activating Th cells. **c** DCs can cross-present MHC-II molecules to CD8+ T cells through the MHC-I pathway and induce their differentiation into CTL. **d** APC and Th cells can produce a variety of co-stimulatory molecules, which provide a second signal for T cell activation, which allows T cells to fully activate

The immunological mechanism of HNCs includes innate immunity and specific immunity mediated by T cells [60]. Innate immunity is the first line of defense against cancer, which includes the direct antitumor effects of NK cells and macrophages. Furthermore, Elmusrati et al. reviewed a large body of literature and concluded that NK cells can further trigger adaptive immune responses by releasing cytokines (IL-2, IL-12, IL-15, IFN-γ, and TNF-α) [61, 62]. However, innate immunity is of limited use against tumors and does not establish long-term immune memory. On the contrary, T-cell-mediated specific immunity plays a key role in this regard. The antitumor effect mediated by T cells in HNCs is the most important mechanism of the antitumor effect in the body (Fig. 2). First, T cells must complete HNC recognition to exert effective immune activity. After complete recognition of HNCs, T cells in the activation process itself, including MHC, provide the first signal to trigger the activation of T cells, and at the same time, of mature APC cells. Th release all kinds of stimulating molecules, thus providing the second signal for activation of T cells, which allows the T cells to fully activate [63]. After complete activation of T cells, a variety of cytokines secreted by APC will continue to participate in the proliferation and differentiation of T cells acting as the third signal required for activation of T cells. Without the participation of such molecules, T cells will undergo apoptosis after activation, and thus interfering and interrupting the overall immune response[64]. After T cells complete their own activation, the antitumor effect mediated by T cells begins formally, and T cells will differentiate into CTL and Th cells to participate in the subsequent immune response.

### Recognition of HNCs by T cells

Every cell in the body has an identity card that determines whether it is allowed to exist or be eliminated, and the MHC gives cells the ability to be specifically recognized by immune cells. In the human body, MHC is collectively known as the human leukocyte antigen (HLA) complex. HLA is a key component of T cell activation that can present captured tumor antigens (TAs) to T cells [65]. Compared with normal cells, HNCs often achieve immune evasion by downregulating HLA expression, so MHC plays a key role in the immune recognition of HNC cells by T cells [66, 67].

#### MHC I recognizes T cells on the surface of HNCs cells

HNCs surface expression of MHC I is an important factor in the specific recognition of tumor by T cells.MHC class I molecules are distributed on the surface of all nucleated cells, and they can recognize and present endogenous antigenic peptides, providing the first signal for T cell activation, which can only initially activate T cells. To study the specific relationship between MHC-I and T cells, Agerer et al. found that the stability of the MHC-I peptide on the cell surface of the SARS-COV-2 gene mutation was decreased, so these cells had a certain ability to evade recognition of CTL [68]. The expression of MHC-I is often dysregulated in tumor cells. Yamamoto et al. found that in pancreatic ductal carcinoma (PDAC), the expression of MHC-I was often inhibited by autophagosomes and lysosomes[69], and in autophagy deficient mice, the expression of MHC-I increased in PDAC cells and T cells produced a stronger antitumor effect[68]. Similarly, HNCs often evade immune monitoring by downregulating class I MHC molecules. Yoo et al. analyzed MHC class I expression in 163 patients with HNC, and immunohistochemistry showed that the expression of MHC class I molecules was significantly reduced in approximately 50% of patients' tumors, among which MHC I expression was not detected in 12.7% of patients[70].

#### T cells are recognized by MHC II presented by APC

Class II molecules are only expressed on the surface of specific cells, such as specialized antigen-presenting cells (B cells, giant cells, DCs), thymic epithelial cells, and activated T cells[71–73]. Specialized antigen-presenting cells (APCs) play the most vital role. These cells can recognize and present exogenous antigenic peptides and present them to T lymphocytes in the form of a molecular complex of antigenic peptide-MHC to activate them in Th cells [74–76], thus inducing activation and proliferation of CD8+ T cells[77]. The recognition function of APC depends on a special receptor expressed on its surface, namely the pattern recognition receptor (PRR). PRRs recognize conserved repetitive gene sequences in microbial species, known as pathogen-associated molecular patterns (PAMPs), and enable APCs to distinguish between themselves, that is, non-self cells and even tumor cells with abnormal gene expression [78, 79]. APC constitutively expresses MHC class II costimulatory molecules and adhesion molecules, which provide initiation signals for T cell activation. The presence of APC provides a powerful aid for tumor antigen presentation and T cell activation.

### Self-activation of T cells in HNCs

After initial recognition of the tumor antigen, T cells need their own activation to produce the antitumor effect. T cell activation needs to receive two types of signals, among which CD8+ T cells specifically recognize MHC-I molecules bound on the surface of HNC. After receiving the first signal, CD8+ T cells activate transcription factors to cause the transcription of various membrane molecules and molecular genes related to cell activation, so they are initially activated as CTL cells. At the same time, APC in contact with T cells are also activated, and MHC-II molecules are presented to CD4+ T cells through the MHC-II pathway to activate Th cells and generate costimulatory molecules to provide a second signal for complete activation of CTL cells.

#### T cells form preliminary activation through the first signal

Specific binding of the T cell antigen receptor (TCR) on the surface of T cells in HNCs and pMHC presented by APC is called antigen recognition, through which T cells initiate their initial activation [80]. Antigen recognition follows MHC restriction, that is, TCR must recognize its own MHC molecules in the pMHC complex while specifically recognizing the antigenic peptides presented by APC. The restriction of MHC restriction determines that any T cell recognizes only the pMHC presented by APC of the same individual. This restriction is determined by the CD4 and CD8 receptors on the surface of T cells. As co-receptors of TCR, CD4+ or CD8+ cells can recognize MHC-II molecules on the surface of tumor cells or APC [71], respectively, and these two molecules can act as the first signal of T cell activation to activate T cells through the MHC-I pathway. CD8+ T specifically recognizes MHC-I on the surface of tumor cells through the MHC-I pathway and is initially activated in cytotoxic T cells (TCL) with a certain activity [81], which is an endogenous recognition pathway that can occur in all nucleated cells. Although the MHC-I pathway only targets endogenous antigens, there is a cross-presenting antigen presentation pathway that can load MHC-II molecules from the exogenous antigen-activated MHC-II pathway into the endogenous MHC-I pathway [82]. This latter pathway plays a key role in antimicrobial and antitumor immunity and immune tolerance [83].

#### T cells complete activation through a second signal

T cells cannot perform tumor clearing without the presence of the second signal.Lafferty et al. found that foreign tissues depleted of white blood cells could not induce an immune response. Furthermore, in the absence of cells that present antigens or immobilized antigens, stimulation of T cells to neither to clonal expansion nor to the production of the IL-2 required to maintain T cell proliferation, leading to the conclusion that T cell activation requires a second signal in addition to the first signal [84]. Meissner et al. compared MHC class II antigen processing machinery (APM) expression in HNCs cell lines (cancer lesions) with interferon (IFN)-gamma-regulated expression profiles and found that there was a lack of constitutive MHC II surface expression in head and neck cancer cellsm[71].Gameiro et al. found that the significant increase in the expression of the MHC II gene was strongly associated with a significant up-regulation of genes for various costimulatory molecules of T cells required for T cell activation and survival after TCGA of more than 500 HNCs [85]. At the same time, Axelrod et al. summarized a large number of studies and concluded that CD4+ T cells can recognize MHC-II and activate them to proliferate as helper T cells (Th) and express the corresponding cytokines involved in the activation of T cells [73]. Homet et al. treated melanin mice with anti-PD-1 and found that recovery of the CTL response required the participation of mouse CD4+ T cells [86]. Meanwhile, Laidlaw et al. concluded that CD8+ T cells could not produce an effective and lasting memory response without the help of CD4+ T cells [87]. Therefore, co-stimulatory molecules with immune-regulating effects can be used as a second signal to participate in complete activation of T cells and to maintain the proliferation and cloning of CTL cells, and MHC-II and CD4+ T cells must be involved in this process.

#### T cells are activated by dual signals provided by DC cells.

Dendritic cells(DC) are very important targets in HNCs immunotherapy. Abolhalaj et al. compared the frequency of DCs in a large number of benign tonsil tissues with that in malignant tonsil tissues. The results showed that the frequency of DCs, the expression of immune molecules and the level of genes involved in immune response were lower in the malignant tonsil group[88].DCs are the most powerful APCs with many maturing dendritic processes, which can recognize, absorb and process exogenous antigens and present antigenic peptides to primary T cells to induce activation and proliferation of T cells [89, 90]. Among APCs, DCs are the only specialized APCs that can capture and process tumor antigens and directly activate primary T cells [91]. DCs provide primary and secondary signals for T cell activation [92]. Immature DCs recognize tumor antigens through PRRs and induce DC maturation and proliferation [92]. Mature DCs present MHC II molecules to CD8+ T cells through cross-presenting [93]. An antigenic stimulus signal (the first signal) that provides initial T cell activation leads to initial T cell activation into CTLs [94]. At the same time, mature DC cells also release multiple exosomes of secretory or paracrine cytokines (second signal) exosomes, which further bind and induce the proliferation and differentiation of activated T cells, thus triggering the initial immune response. In addition, DCs have a certain immunomodulatory effect. DCs secrete a large amount of IL-I2 to induce primary T cells (Th0) that differentiate into Th1 cells and produce the immune response Th1 cell. However, it is not clear how cross-presenting allows CD8+ T cells to activate recognition of all antigenic peptides presented by exogenous cells. DC cells are excellent mediators, which can recognize tumor antigens and present two signals required for T cell activation at the same time. Therefore, HNCs tumor vaccine prepared based on DC cells has been widely used in clinic.

### T cell-mediated adaptive immune mechanism of HNCs

Schumacher et al. reviewed a large number of studies and concluded that tumor cells have different antigen components from normal tissue cells, and it is by recognizing these antigens that T cells form specific killing effects on tumor cells [81]. Tumor cells can escape normal immune monitoring because most are weakly immunogenic, and it is difficult for the body to induce a specific immune responses to these antigens. However, as an existing immune mechanism in the body, artificially inducing and activating this pathway to recognize and kill tumor cells is a means of immunity with the least damage to the body. Among them, tumor immunotherapy related to T cells has become the focus of immunotherapy.

#### T cells are induced to differentiate into cytotoxic T cells to kill HNC cells

CTL is a key component of antitumor immunity [95]. A large amount of evidence showed that the depth of CTL infiltration in the body was closely related to the prognosis of HNCsS [15, 19, 96]. Apoptotic or necrotic tumor cells release antigens, which are processed by APC, including DC, and present MHC to TCR receptors in CD8+ T cells. When the tumor releases a large number of highly expressed costimulatory molecules of EVs, APCs or DCs can directly present the antigen to CD8+ T cells[97], stimulate their synthesis of IL-2 and proliferation and differentiation into CTL with a specific killing effect on tumor cells. CTL specifically kills target cells by recognizing pMHC-I presented by target cells. However, HNCs often avoid CTL recognition by reducing the expression of MHC I, thus forming immune evasion. The killing mechanism of CTL on tumor cell. includes the following two pathways: one is the perforin/granzyme pathway and the other is the death receptor pathway [98]. Stimulation of CTL initiation is mediated in the form of cytokines secreted by CD4+ Th cells [99]. CD4+ T cells interact with antigens in the MHC-II molecular pathway and secrete cytokines to help CTL proliferation and activation. Therefore, when the tumor does not express or has a low expression of costimulatory molecules, the activation of CD8+ T cells also requires the help of activated CD4+ Th cells [25]. CTL's killing of HNCs depends on a variety of activation signals. However, tumor cells often reduce or inhibit the expression of relevant activation signals to avoid CTL's killing. At present, most T cell-related immunotherapy aims to up-regulate relevant activation signals to enhance the killing and recognition ability of CTL cells to tumors.

#### T cells induce differentiation into Th cells to regulate the killing effect of CTL HNCs

In mice, CD4+ Th1 cells play an important auxiliary role in the activation of CD8+ CTL [100]. When the body has low expression of costimulatory molecules, these can be secreted by Th cells to activate CD8+ T cells. While inducing CTL differentiation, some CD4+ Th1 cells also have the ability to kill tumor cells directly [101]. To date, the role of CD4+ Th in HNCs immunotherapy is obviously neglected. The immune response of T cells is always suboptimal without the help of CD4+ Th cells [102]. However, the exact role of Th cells in HNCs remains unclear [19]. There is still a gap in Th cell related HNCs immunotherapy.

## Immunotherapy of HNCs associated with T cells

Many of the difficulties in studying and treating HNCs are that HNCs are a heterogeneous group of cancers of different anatomical structures, are highly immunosuppressive [103], are associated with different risk factors, and have different molecular pathology. Despite current state-of-the-art treatments, the recurrence rate of HNCs remains unacceptably high [104]. In fact, 65% of HNCs recur [105], and in these cases, the 5-year survival rate for patients with advanced HNC is only 35% to 45% [106, 107]. To eliminate this adverse outcome, the treatment of HNCs has begun to adopt immunotherapy strategies [108]. T lymphocytes as the main antitumor cells, most tumor immunotherapy methods seek to expand cytotoxic T lymphocytes targeting malignant cells [30]. Currently, antitumor vaccines, targeted drugs, immune checkpoint inhibitors, and other therapies have been widely used in HNC immunotherapy (Table 1).

**Table 1:** Progress in immunotherapy protocols for HNCs

Treatment	Mechanisms mediated by T cells	Advantages	Disadvantages	Reference(s)
Tumor vaccine	T cells are activated directly by HNC-specific antigens	Almost no toxicity	Difficult to develop and achieves no definitive effect on the most advanced patients	[109, 110]
Targeted drugs	NK cells, which induce T cells, produce immune effects	An essential part of combination therapy	Use alone does not significantly improve survival in patients with advanced and R/M HNCs	[111]
CAR-T	Activated tumor antigen-specific T cells are directly injected into the host	The immune effect is effective and long-lasting	Dependent on the harsh TME Application in HNC remains to be seen	[27]
ICIs	Regulation of T cell activity	The effects are long-lasting	Do not significantly improve survival in patients with advanced and R/M HNCs	[112]
Combined immunotherapy	Novel immunomodulators bind to PD1/PD-L1 inhibitors	Can significantly improve the remission rate of patients compared to immunotherapy alone	Still to be seen	[113]

### Specific immune effects of tumor vaccine-activated T cells on HNCs

Tumor vaccines are an immune-mediated therapy aimed at inducing an antitumor immune response by injecting immunogenic tumor vaccine into the host. This is a targeted immunotherapy. Currently, protein polypeptide vaccines, gene modified vaccines, and DC vaccines have the most developed. Cancer vaccines are designed to activate T cells to generate an adaptive immune system by presenting the tumor antigen TSA or TAA via APC. TSA-specific vaccines are preferred because the antigen will be specific to tumor cells, thereby avoiding an autoimmune response to normal cells [114]. TSA-targeted vaccines may be suitable for only a small subset of HNCs because HNCs, particularly HPV-negative HNCs, have a high mutation rate [115]. DC cells have unique functions in absorbing tumor antigens and activating T cells to produce antitumor effects, so the DC vaccine is a very potential tumor vaccine therapy among many vaccines [89]. Gross et al. have shown that DC vaccines are safe for several types of cancer and have been shown in clinical trials to induce long-lasting T cell responses[116]. Schuler et al. and Whiteside et al. tested toxicity and efficacy in HNC patients using peptides synthesized from autologous DCS in vitro or vaccines loaded with tumor protegens or tumor cells. They found that these drugs were not toxic, but the preparation was laborious and, despite repeated use in patients with advanced HNC, most were ineffective [109, 110]. Whiteside et al. concluded that tumor vaccine inoculation for HNCS patients could produce a certain immune effect on the body, but the therapeutic effect on the tumor was still not obvious. In a word, tumor vaccine can be used as an auxiliary means in the combination of HNCS and preventive therapy, which can effectively stabilize TME, provide a good environment for other treatments, and prevent tumor recurrence to a certain extent[117].

### Antitumor effects of T cells induced by targeted drugs

Cetuximab is one of the best targeted drugs for head and neck tumors, and its main antitumor effect is attributed to the blocking of EGFR signal[118].Gildener-Leapman et al. found that epidermal growth factor (EGFR) was overexpressed in 80%-90% of HNC cases, resulting in excessive proliferation, invasion, and angiogenesis of tumor cells [119]. Cetuximab is a monoclonal antibody of chimeric immunoglobulin G1 (IgG1) [120] that competently inhibits ligand binding to EGFR and induces immune function. For example, activation of the FC receptor FCgRIIIa on the surface of NK cells induces ADC, or cross-activation of DCs and NK stimulates cytotoxic T cells to form an antitumor response [121].

The landmark EXTREME trial, published in 2008, evaluated cetuximab in 442 patients with HNC. The results showed a median survival increase of 2.7 months in the cetuximab group compared to those treated with chemotherapy alone [111]. Cetuximab was approved by the US FDA in November 2011 for the first-line treatment of R/M HNC [122]. In 2006, Bonner et al., in a study evaluating advanced local HNCs, found that adding cetuximab to RT could improve the local control rate of tumors and could significantly improve OS [123]. Gillison and Gebre et al., through subsequent phase II and III studies, showed that cisplatin combined with RT is superior to cetuximab plus RT in the local control of HNCs and improvement of OS [124, 125]. Mehanna et al. conducted an open-label randomized controlled Phase 3 trial on patients with low-risk papillomavirus-positive oropharyngeal cancer, and found that cetuximab showed no advantage in reducing drug toxicity and improving patient survival compared with cisplatin protocols. And there is some damage to tumor control[126]. There is increasing evidence that cetuximab alone is not a recommended strategy for treating head and neck tumors.Today, cetuximab is often used in combination with chemoradiotherapy and immune checkpoint inhibitors, which provides a direction for future combination immunotherapy [127–129].

### T cells can be used directly as immunotherapy drugs for HNCs: CAR-T

T cells amplified and activated in vitro, including cytokine-induced killer cells (CIK), TIL, and tumor antigen-specific CTL, can also be adopted into the tumor-bearing host. The most important achievement in this regard has been modified T cells based on chimeric antigen receptor (CAR) (CAR-T) [130]. In CAR T cell therapy, genetically engineered autologous T cells expressing CAR are used in patients. CAR binds antigens independently of MHC and is therefore immune to downregulation of MHC of tumor cells, which is an effective means of avoiding immune evasion by malignant tumor cells [131]. However, this approach has obvious defects, that is, side effects caused by cytokine release lead to life-threatening immune overactivation and neurotoxicity, and the treatment effect of this therapy in solid tumors is not satisfactory [132, 133]. In the treatment of HNCs, several CAR T cells targeting different antigens have been developed and entered clinical trials, including ErbB, HER2, MUC1, LMP1, NKG2D, etc. One of the attractive antigens is the ErbB family, which consists of four members called epidermal growth factor receptors EGFR or erBB-1, erBB-2, erBB-3, erBB-4. Larcombe-Young et al. found that dysregulation of ErbB signaling was common in the pathogenesis of HNCS, and EGFR was strongly overexpressed in 90% of the cases[134]. However, targeted therapy for EGFR is often influenced by members of the ErbB family, leading to a poor prognosis. Pan-targeted ErbB T4 + CAR therapy for these conditions has entered clinical trials. CAR + T4 was specific against all EGFR homologous ErbB antigens simultaneously. In this way, T cells obtained strong cytotoxic activity against multiple tumor types, reducing the impact of homologous tumor-associated antigens on CAR-T therapy. Meanwhile, a safety clinical trial of T4 treatment in HNCS patients (NCT01818323) showed that no cytotoxic events occurred in the majority of patients receiving T4 continuous treatment[135]. However, the treatment of CAR-T in head and neck tumors still faces challenges such as the transport and osmosis of CAR-expressed T cells into the tumor microenvironment of malignant deposition, malignant inflammation, hypoxia, and metabolic dysfunction[60]. Therefore, it is not difficult to find that CAR-T therapy depends on the tightly controlled tumor microenvironment, which may give CAR-T therapy in combination therapy mode and may show better efficacy.

### Examination of immune checkpoint inhibitors to improve the immune effect of T cells on HNCs

Removal of the immunosuppressive state of tumor patients to treat tumors is the biggest breakthrough in the theory and application of tumor immunotherapy; the most prominent progress is immune checkpoint therapy. Immune checkpoint molecules are a class of immunosuppressive molecules such as cytotoxic T lymphocyte-associated protein 4 (CTLA-4) and Programmed death protein 1 (PD-1) [136], which are inhibitory costimulatory molecules that act after activation of T lymphocytes and persist in the presence of stimulus. Pd-1 is often seen as a "depletion marker" whose stimulation induces the formation of impotent T cells [137]. These cells do not express adjuvant (CD4+) or cytotoxic (CD8+) activity against tumor cells [138]. Zandberg and Strome et al. evaluated PD-1 staining results of HNCs (mainly oropharyngeal tumors) and showed that approximately half of oropharyngeal squamous cell carcinomas (OSCCs) expressed PD-L1 [139]. A higher expression of PD-1 was identified in HPV(+) tumors. Immune checkpoint therapy is a therapeutic strategy that modulates T cell activity to improve the tumor immune response by targeting co-inhibitory or co-stimulating signals. PD-1 and CTLA-4 are thought to be marker genes for depleted CD8+ T cells [140]. The study of CTLA-4 and PD-1 and its PD-1 ligand is considered a milestone event in immunotherapy.

In 2016, nivolumab and pembrolizumab, two anti-PD-1 antibodies, were shown to improve survival (OS) in patients with relapsed metastatic HNC and were approved by the US Food and Drug Administration (FDA) for second-line therapy [141]. This provides a new standard of care for R/M HNCs. Compared to standard cytotoxic chemotherapy, immunotherapy improves patient survival through a durable response [142]. Although this is an encouraging development, several studies have shown that while the PD-1/PD-L1 inhibitor in HNC immunotherapy can increase the response rate from 13 to 20%, survival (OS) improves in only 1 in 10 patients. This statistical result may indicate that few patients with HNC respond to immune checkpoint therapy[143], but that a certain percentage of patients will have lasting benefits [112].

### Combination immunotherapy associated with T cells in HNCs

David et al.'s Version 2.2020, NCCN Clinical Practice Guidelines in Oncology indicate that the current preferred nonsurgical treatment for HNCs is platinum-based dual chemotherapy (mainly cisplatin and carboplatin) [144]. Refractory and metastatic HNCs are often targeted with drugs such as cetuximab, methotrexate, and taxanes, all of which have significant side effects and a low response rate of only 10–13% [145]. Chemotherapy in HNC patients tends to be temporary, does not significantly extend life expectancy, and rarely prevents most patients from dying of malignancy [146]. Based on the above conclusions, it is not difficult to find that the efficacy of antiviral drugs alone for patients with HNC is limited. Therefore, international research on immunocombination therapy is increasing, that is, the combination of a novel immune modulator with PD1/PD-L1 inhibitors [147]. Chemotherapeutic agents are logical partners of ICIs in HNC. ICIs improve cytotoxic T cells and promote tumor regression and immune rejection. Targeted drugs can induce antibody-dependent cytotoxicity (ADC) and cause immune cells to talk to each other, including NK cells and DCs. This mutually reinforcing immune effect can initiate tumor antigen-specific cellular immunity and enhance the antigen-specific T-cell response [148].

Multiple trials have also shown that multimodal combinations of treatments have better response rates than conventional therapies alone[113]. Burtness et al. reported that pembrolizumab combined with platinum and 5-fluorouracil significantly improved OS in 882 patients with locally untreatable recurrent or metastatic HNC treated at 200 centers in 37 countries, compared to cetuximab plus platinum and 5-fluorouracil alone [149]. In 33 patients with platinum-resistant recurrent or metastatic HNC (platinum-resistant, recurrence, or metastatic HNCs), Sacco et al. demonstrated that pembrolizumab combined with cetuximab could achieve an overall response rate of 45%. This exceeds the published response rates for pembrolizumab (16–18%) or cetuximab (6–13%). Furthermore, the overall response rate was consistent with that of pembrolizumab combined with platinum bivalent chemotherapy (36–43%) in Burtaness et al. Effective evaluation of the large number of immune combinations in HNC still requires more innovative experimental design and analysis of the biomarkers of the serum [150]. We can foresee the development and introduction of HNCs immunotherapy in the future as a powerful complement to traditional therapies (surgery, RT, and chemotherapy).

HNCS often have a complex tumor microenvironment, and single immunotherapy is often affected by a variety of unpredictable factors, including inflammation, hypoxia and metabolic disorders in the tumor microenvironment. Combination therapy can ensure that the tumor is in a stable or even beneficial state of immune effect of immune drugs in many aspects, and there are even complementary effects between multiple drugs. We can foresee the development and introduction of HNCs immunotherapy in the near future, which is expected to be a powerful complement to traditional therapies (surgery, RT and chemotherapy).

## Conclusions

Traditional surgical treatment has significant detrimental impact on the quality of life of HNC patients, while for R/M HNC patients, the effect of traditional surgical treatment and radiation therapy and chemotherapy is poor and often cannot significantly improve the survival rate of patients. Therefore, immunotherapy is necessary for HNC patients. Several studies have shown that T cells are the main antitumor cells. However, HNCs can reduce MHC expression in many ways to avoid recognition and killing of T cells. Therefore, how to inhibit immune escape from tumor cells or improve the immune effect of T cells on tumor cells is now the focus of immunotherapy research. Understanding and clarifying the mechanism of T cells with antitumor activity in the body can be targeted to develop relevant immunological treatment methods. However, in HNC immunotherapy, the effect of single immunotherapy is not ideal and is characterized by two concurrent problems: an exceptionally low response rate and a remarkably high recurrence rate. The emergence of combined immunotherapy provides a new direction and hope for HNC immunotherapy. Especially combination immunotherapy of ICIs and targeted drugs theoretically should induce synergistic effects and the efficacy of this combination has been evidenced in clinical trials.

## Data Availability

Not applicable.

## Abbreviations

HNCs: Head and neck tumors
CTL: Cytotoxic T cells
Th: Helper T cells
ICI: Immune checkpoint inhibitor
HPV: Human papillomavirus
R/M HNCs: Recurrent/metastatic head and neck tumors
RT: Radiotherapy
Tregs: Regulatory T cells
APCs: Antigen presenting cells
EV: Extracelluar vesicles
CRT: Chemotherapy
TIL: Tumor-infiltrating lymphocytes
DC: Dendritic cell
HLA: Human leukocyte antigen
TAs: Tumor antigens
PDAC: Pancreatic ductal carcinoma
PRR: Pattern recognition receptor
PAMPs: Pathogen-associated molecular patterns
TCR: T cell antigen receptor
EGFR: Epidermal growth factor
CIK: Cytokine-induced killer cells
CTLA-4: Lymphocyte-associated protein 4
OSCCs: Oropharyngeal squamous cell carcinomas
ADC: Antibody-dependent cytotoxicity
NK: Natural killer
TSA: Tumor specific antigens
TAA: Tumor-associated antigens
IL: Interleukin
IFN: Interferon
TNF: Tumor necrosis factor
CAR-T: Chimeric antigen receptor T cells
PD1: Programmed cell death 1
PD-L1: Programmed death-ligand 1
